# Journalistic Change

**Published:** 1883-07

**Authors:** 


JOURNALISTIC CHANGE.—The *Missouri Dental Journal*, which has been published ever since its establishment at St., Louis, owing to a transfer of ownership, is now published at Kansas City. Dr. C. W. Spalding retires from the editorship after an occupancy of five years, his place being supplied by Dr. R. J. Pearson, as managing editor, and Drs. J. D. Patterson and C. L. Hungerford, as assistants.

We note with regret the withdrawal of Dr. Spalding, who has proven himself to be an able editor, and extend to the new organization our best wishes for an increased circulation, and success in their new field of labor.

